# Metabolic syndrome in patients with type 2 diabetes and atherosclerotic cardiovascular disease: a post hoc analyses of the EMPA-REG OUTCOME trial

**DOI:** 10.1186/s12933-020-01174-6

**Published:** 2020-11-26

**Authors:** João Pedro Ferreira, Subodh Verma, David Fitchett, Anne Pernille Ofstad, Sabine Lauer, Isabella Zwiener, Jyothis George, Christoph Wanner, Bernard Zinman, Silvio E. Inzucchi

**Affiliations:** 1 Centre d’Investigations Cliniques Plurithématique Inserm 1433, CHRU de Nancy, Inserm U1116, FCRIN INI- CRCT, Université de Lorraine, Nancy, France; 2 Division of Cardiac Surgery, St Michael’s Hospital, University of Toronto, Toronto, ON Canada; 3 Division of Cardiology, St Michael’s Hospital, University of Toronto, Toronto, ON Canada; 4 Boehringer Ingelheim Norway Ks, Asker, Norway; 5 Boehringer Ingelheim Pharma GmbH & Co. KG, Ingelheim, Germany; 6 Boehringer Ingelheim International GmbH, Ingelheim, Germany; 7 Würzburg University Clinic, Würzburg, Germany; 8 Mount Sinai Hospital, Lunenfeld-Tanenbaum Research Institute, University of Toronto, Toronto, ON Canada; 9 Yale University School of Medicine, New Haven, CT USA

**Keywords:** Type 2 diabetes mellitus, Metabolic syndrome, Empagliflozin, Treatment effect, Cardiovascular disease

## Abstract

**Background:**

Patients with type 2 diabetes (T2D) and metabolic syndrome (MetS) are at greater cardiovascular risk than those with T2D without MetS. In the current report we aim to study the characteristics, cardio-renal outcomes and the effect of empagliflozin in patients with MetS enrolled in the EMPA-REG OUTCOME trial.

**Methods:**

A total of 7020 patients with T2D and atherosclerotic cardiovascular disease were treated with empagliflozin (10 mg or 25 mg) or placebo for a median of 3.1 years. The World Health Organization MetS criteria could be determined for 6985 (99.5%) patients. We assessed the association between baseline MetS and multiple cardio-renal endpoints using Cox regression models, and we studied the change in the individual component over time of the MetS using mixed effect models.

**Results:**

MetS at baseline was present in 5740 (82%) patients; these were more often white and had more often albuminuria and heart failure, had lower eGFR and HDL-cholesterol, and higher blood pressure, body mass index, waist circumference, and triglycerides. In the placebo group, patients with MetS had a higher risk of all outcomes including cardiovascular death: HR = 1.73 (95% CI 1.01–2.98), heart failure hospitalization: HR = 2.64 (95% CI 1.22, 5.72), and new or worsening nephropathy: HR = 3.11 (95% CI 2.17–4.46). The beneficial effect of empagliflozin was consistent on all cardio-renal outcomes regardless of presence of MetS.

**Conclusions:**

A large proportion of the EMPA-REG OUTCOME population fulfills the criteria for MetS. Those with MetS had increased risk of adverse cardio-renal outcomes. Compared with placebo, empagliflozin improved cardio-renal outcomes in patients with and without MetS.

*Trial registration* Clinical Trial Registration: URL: https://www.clinicaltrials.gov. Unique identifier: NCT 01131676

## Introduction

Metabolic syndrome (MetS) comprises interrelated risk factors that, together, contribute to a cumulative risk of cardiovascular (CV) complications [1]. The World Health Organization (WHO) defines MetS by the presence of insulin resistance [e.g. type 2 diabetes (T2D) or indications of abnormal glucose metabolism], together with at least two of the following factors: use of anti-hypertensive medication and/or high blood pressure (BP) ≥ 140 mmHg systolic or ≥ 90 mmHg diastolic], plasma triglycerides > 150 mg/dL, HDL cholesterol < 35 mg/dL in men or < 39 mg/dL in women, body mass index (BMI) > 30 kg/m^2^ and/or waist-hip ratio > 0.9 in men, > 0.85 in women, and urinary albumin excretion rate ≥ 20 µg/min or albumin-creatinine ratio (UACR) ≥ 3.4 mg/mmol [2]. A recent report demonstrated that even among patients with diabetes, those with uncontrolled components of the MetS, are at higher risk for adverse CV outcomes [3]. Therefore, improving any or several of the factors defining MetS may improve CV outcomes in patients with diabetes.

In the EMPA-REG OUTCOME trial [4], the SGLT2 inhibitor empagliflozin reduced the risk of CV death, hospitalization for heart failure (HF) and incident or worsening nephropathy compared with placebo in patients with T2D and atherosclerotic CV disease (ASCVD). Empagliflozin also reduced HbA1c, BP, body weight and UACR [5–8]. To extend these findings, we sought to explore whether the MetS confers higher risk of events in patients with diabetes and ASCVD, if empagliflozin can positively impact the individual components of the MetS, and whether the cardiorenal benefits of empagliflozin extended equally to those with vs. without MetS.

## Materials and methods

### Study design

The design of EMPA-REG OUTCOME has been described previously [4]. Briefly, the study population comprised patients with type 2 diabetes, established ASCVD, HbA1c 7.0–9.0% for drug-naïve patients and 7.0–10.0% for those on stable glucose-lowering therapy, and an estimated glomerular filtration rate (eGFR) [determined by the Modification of Diet in Renal Disease (MDRD) equation] ≥ 30 mL/min/1.73 m^2^. Patients were randomized 1:1:1 to receive empagliflozin 10 mg, empagliflozin 25 mg, or placebo in addition to standard of care. The first 12 weeks, blood glucose lowering treatment was to be kept unchanged. Thereafter and throughout the trial, investigators were encouraged to adjust diabetes medication and treat cardiovascular risk factors to achieve optimal standard of care according to local guidelines. The trial was to continue until ≥ 691 patients had experienced an adjudicated event included in the primary outcome [3-point major adverse CV events (3P-MACE): CV death, non-fatal myocardial infarction (MI), or non-fatal stroke].

### Outcomes

In the current analyses we explore CV death, hospitalization for HF, CV death or hospitalization for HF (excluding fatal stroke), all-cause mortality, 3P-MACE, and incident or worsening nephropathy defined as progression to macroalbuminuria, doubling of the serum creatinine level accompanied by an eGFR of ≤ 45 ml per minute per 1.73 m2, initiation of continuous renal-replacement therapy, or death from renal disease [4, 8]. All cardiovascular outcome events and deaths were prospectively adjudicated by two clinical events committees (for cardiac and neurological events).

### Definition of metabolic syndrome

We used the WHO definition of MetS (2) where the first criteria (insulin resistance or diabetes) was present in all patients. Urinary albumin excretion rate and waist/hip-ratio were not assessed in the trial. Hence, at least two criteria of the following needed to be fulfilled to qualify a patient as having MetS: (1) use of antihypertensive medication and/or high BP (≥ 140 mmHg systolic or ≥ 90 mmHg diastolic), (2) plasma triglycerides > 150 mg/dL (≥ 1.7 mmol/L), (3) HDL cholesterol < 35 mg/dL (< 0.9 mmol/L) in men or < 39 mg/dL (< 1.0 mmol/L) in women, (4) BMI > 30 kg/m^2^, and (5) UACR ≥ 3.4 mg/mmol. In the current analyses, we applied the cut-offs as given in mmol/L. Hence, this post-hoc analysis includes patients with T2D and if two additional features of the MetS are present these patients are classified as having T2D and MetS, referred to as “with MetS” throughout the manuscript and tables.

### Statistical analyses

All analyses were undertaken in patients that were treated with at least one dose of study drug and had baseline information for assessment of MetS available. Descriptive data are given as mean ± standard deviation, or proportions (%). We split the population into two subgroups (with vs. without MetS at baseline) based on whether the criteria for MetS were fulfilled or not. Incidence rates per 1000 patient years of follow-up were calculated in the placebo group. The association of subgroup to outcomes and the treatment effect of pooled empagliflozin vs. placebo was explored by Cox proportional hazards models with terms for age, sex, geographic region, baseline HbA1c, baseline eGFR, treatment, subgroup and subgroup*treatment interaction. In addition, we assessed if there was any treatment interaction with subgroups based on how many of the diagnostic criteria for MetS that were fulfilled among those with MetS at baseline using the same Cox model. Treatment effects on the separate components of MetS [HbA1c, systolic BP, weight, waist circumference (WC), HDL-cholesterol, triglycerides, and UACR] were evaluated using mixed effect model repeat measurement (MMRM) models. The models included subject as a random effect, and baseline HbA1c plus the baseline value of the MetS component being explored as linear covariates along with their interaction with visit time (weeks). Additionally, baseline eGFR, baseline BMI and geographic region were also used as adjustment covariates in the models. The MMRM model also included a fixed categorical effect for ‘time of randomization’ to account for each patient’s theoretical ability to ‘reach’ certain weeks in this study arising from the study design. In addition, the model included the terms visit, treatment and MetS at baseline, as well as all two-and three-way interactions thereof. For UACR, values were assumed to be not normally distributed, such that values were log-transformed prior to analyses. All analyses were performed with SAS version 9.4.

## Results

### Baseline characteristics

Of the 7020 patients randomized and treated in the trial, 6985 (99.5%) had baseline information available to evaluate the MetS criteria. Of these, 5740 (82%) had MetS (1902/3838 in placebo/empagliflozin groups, respectively). Baseline characteristics comparing patients with and without MetS are shown in Table 1. As expected, those with MetS were more often white and had more often albuminuria, had lower eGFR and HDL-cholesterol, and higher blood pressure, BMI, WC, and triglycerides. Furthermore, they used more BP lowering drugs, and had a HF diagnosis more often than those without MetS.

**Table 1:** Baseline characteristics in the treatment groups separately in patients with versus those without metabolic syndrome at baseline

	With metabolic syndrome		Without metabolic syndrome	
	Placebo n = 1902	Empagliflozin n = 3838	Placebo n = 426	Empagliflozin n = 819
Female	546 (28.7)	1119 (29.2)	104 (24.4)	228 (27.8)
Ethnicity				
White	1423 (74.8)	2891 (75.3)	251 (58.9)	491 (60.0)
Black/African American	101 (5.3)	187 (4.9)	19 (4.5)	48 (5.9)
Asian	359 (18.9)	723 (18.8)	151 (35.4)	276 (33.7)
Native Hawaiian	3 (0.2)	5 (0.1)	1 (0.2)	1 (0.1)
American Indian, Alaskan	16 (0.8)	31 (0.8)	4 (0.9)	3 (0.4)
Missing	0	1 (< 0.1)	0	0
Age, years	63.1 ± 8.7	63.1 ± 8.5	63.7 ± 9.1	63.2 ± 8.9
T2D duration, years				
≤ 1	46 (2.4)	106 (2.8)	6 (1.4)	21 (2.6)
> 1 to 5	309 (16.2)	579 (15.1)	61 (14.3)	126 (15.4)
> 5 to 10	474 (24.9)	986 (25.7)	95 (22.3)	186 (22.7)
> 10	1073 (56.4)	2167 (56.5)	264 (62.0)	486 (59.3)
Medication				
Metformin^a^	1415 (74.4)	2840 (74.0)	315 (73.9)	599 (73.1)
Insulin^a^	967 (50.8)	1912 (49.8)	166 (39.0)	324 (39.6)
Beta blocker	1268 (66.7)	2579 (67.2)	227 (53.3)	459 (56.0)
Diuretics	871 (45.8)	1785 (46.5)	116 (27.2)	254 (31.0)
ACEi/ARBs	1571 (82.6)	3200 (83.4)	293 (68.8)	576 (70.3)
Statins	1431 (75.2)	2959 (77.1)	338 (79.3)	646 (78.9)
eGFR, mL/min/1.73 m^2^	72.83 ± 21.25	73.12 ± 21.83	78.25 ± 19.66	78.91 ± 19.88
Prior stroke	448 (23.6)	923 (24.0)	104 (24.4)	155 (18.9)
Prior CAD	1444 (75.9)	2901 (75.6)	315 (73.9)	619 (75.6)
Prior MI	895 (47.1)	1799 (46.9)	186 (43.7)	382 (46.6)
Prior PAD	387 (20.3)	813 (21.2)	90 (21.1)	164 (20.0)
Heart failure	219 (11.5)	399 (10.4)	25 (5.9)	61 (7.4)
Retinopathy	429 (22.6)	856 (22.3)	93 (21.8)	164 (20.0)
BMI, kg/m^2^	31.65 ± 5.17	31.57 ± 5.19	26.28 ± 2.71	26.22 ± 2.83
Weight, kg	89.46 ± 19.13	89.15 ± 18.83	74.15 ± 12.53	72.78 ± 12.30
Waist circumference, cm	107.2 ± 13.8	107.0 ± 13.4	95.2 ± 9.6	94.2 ± 9.5
SBP, mmHg	136.8 ± 17.4	136.4 ± 17.1	131.4 ± 16.0	130.0 ± 15.4
DBP, mmHg	77.3 ± 10.3	77.2 ± 9.8	74.8 ± 9.3	73.9 ± 9.0
HbA1c, %	8.10 ± 0.85	8.10 ± 0.85	7.97 ± 0.80	7.94 ± 0.81
UACR				
Normal (< 30 mg/g)	971 (51.1)	1968 (51.3)	407 (95.5)	803 (98.0)
Micro (30–300 mg/g)	663 (34.9)	1325 (34.5)	12 (2.8)	12 (1.5)
Macro (> 300 mg/g)	254 (13.4)	505 (13.2)	6 (1.4)	4 (0.5)
Missing	14 (0.7)	40 (1.0)	1 (0.2)	0
Uric acid, mg/dL	6.11 ± 1.68	6.05 ± 1.67	5.51 ± 1.50	5.48 ± 1.41
ASAT, U/L	23.07 ± 10.65	22.61 ± 9.81	22.05 ± 8.75	21.93 ± 8.69
ALAT, U/L	26.74 ± 15.85	25.88 ± 14.01	24.13 ± 12.25	23.77 ± 12.47
HDL-C, mg/dL	42.6 ± 10.8	43.3 ± 11.6	50.6 ± 11.3	50.5 ± 11.2
LDL-C, mg/dL	85.6 ± 36.0	86.4 ± 36.6	81.6 ± 32.3	83.6 ± 32.4
Triglycerides, mg/dL	185.8 ± 128.4	185.1 ± 138.1	103.6 ± 35.6	103.6 ± 31.2
Fufilling 2 criteria^b^	730 (38.4)	1500 (39.1)	NA	NA
Fulfilling 3 criteria^b^	661 (34.8)	1332 (34.7)	NA	NA
Fufilling 4 or 5 criteria^b^	478 (25.1)	936 (24.4)	NA	NA
Not evaluable for number of fulfilled criteria^c^	33 (1.7)	77 (2.0)	NA	NA

Data are n (%) or mean ± SD

*ACEi* angiotensin converting enzyme inhibitor*,* *ARB* angiotensin receptor blocker, *ALAT* aspartate amino transferase, *ASAT* aspartate amino transferase, *BMI* body mass index, *CAD* coronary artery disease, *DBP* diastolic blood pressure, *eGFR* estimated glomerular filtration rate, *HbA1c* glycated hemoglobin, *HDL-C* high-density lipoprotein cholesterol, *LDL-C* low-density lipoprotein cholesterol, *MI* myocardial infarction, *PAD* peripheral artery disease, *SBP* systolic blood pressure, *T2D* type 2 diabetes, *UACR* urine albumin-creatinine ratio

^a^Background medication at baseline

^b^Of the additional WHO diagnostic criteria on top of insulin resistance/diabetes

^c^Patients with MetS and 2 or 3 criteria fulfilled but not evaluable for at least one of the remaining criteria

### Cardiorenal, mortality and HF outcomes

**Fig. 1:**
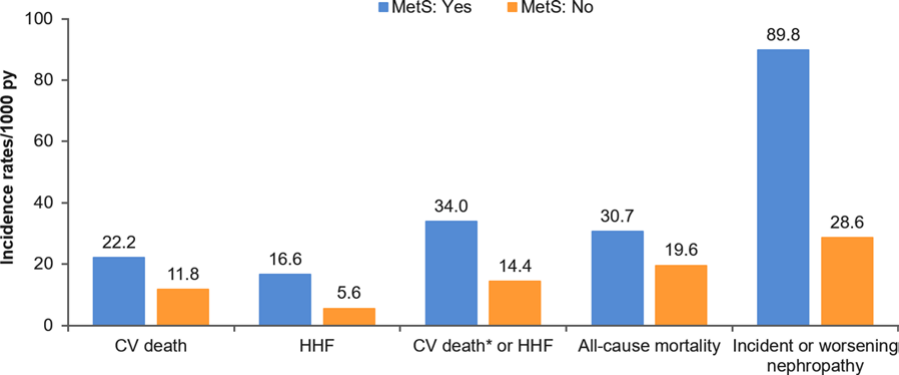
Incidence rates of outcomes in the placebo group in those with versus those without metabolic syndrome at baseline. *Excluding fatal stroke. *CV* cardiovascular, *HHF* hospitalization for HF, *PY* patient years

**Fig. 2:**
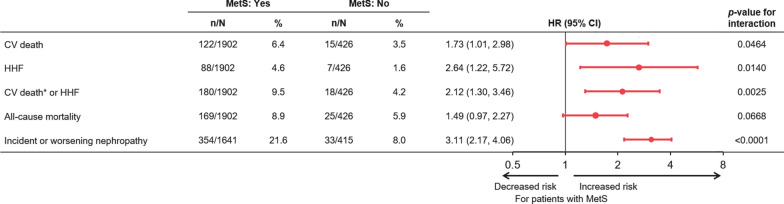
Association of metabolic syndrome at baseline and outcomes in the placebo group (patients without metabolic syndrome is reference group). *Excluding fatal stroke. Cox models include terms for baseline age, sex, HbA1c, eGFR, geographical region, treatment, MetS at baseline and treatment*MetS at baseline interaction. *CV* cardiovascular, *HHF* hospitalization for heart failure, *MetS* metabolic syndrome

**Fig. 3:**
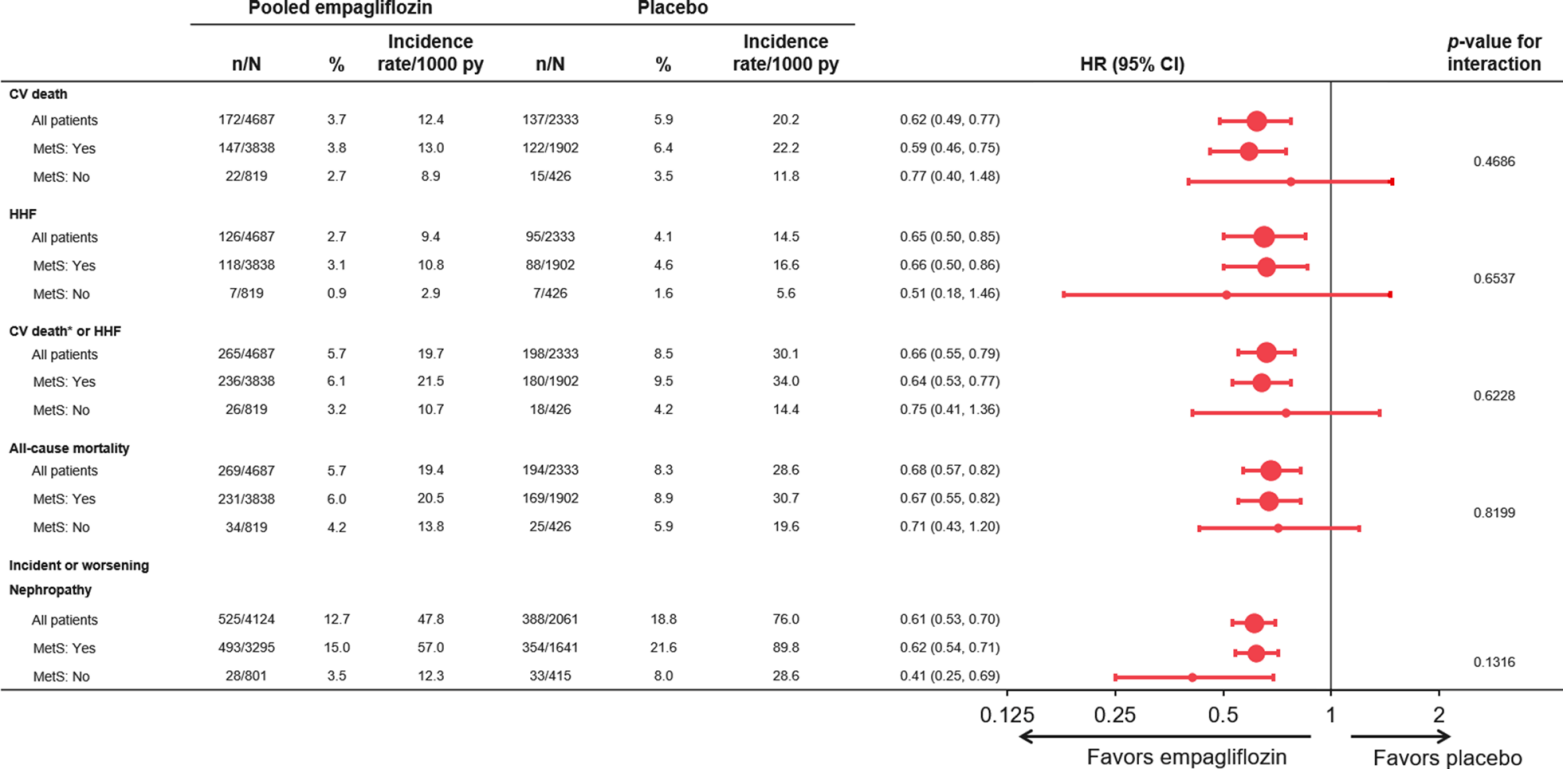
Consistent treatment effect of empagliflozin vs. placebo in those with and without metabolic syndrome at baseline. *Excluding fatal stroke. Cox models include terms for baseline age, sex, HbA1c, eGFR, geographical region, treatment, MetS at baseline and treatment*MetS at baseline interaction. p-values for treatment-by-subgroup interaction were obtained from tests of homogeneity of treatment group differences among subgroups with no adjustment for multiple testing. *CV* cardiovascular, *HHF* hospitalization for heart failure, *MetS* metabolic syndrome, *py* patient-years

Patients with MetS had increased risk of all outcomes with incidence rates in the placebo group of approximately 1.5 to 3-fold higher than the rates observed in patients without MetS in the placebo group (Fig. 1). The increase in risk was highest for incident or worsening nephropathy with a hazard ratio (HR) of 3.11 (2.17, 4.46), p < 0.0001 (Fig. 2). Empagliflozin reduced the risk of all of these outcomes and was consistent in those patients with MetS and without MetS (all interaction p-values > 0.05) (Fig. 3). Additionally, the treatment effect was furthermore consistent in patients with MetS regardless of whether they fulfilled 2, 3 or of the additional diagnostic criteria for MetS (interaction p-value for CV death: =0.24, hospitalization for HF = 0.65, CV death or hospitalization for HF 0.40, all-cause mortality 0.24). For new or worsening nephropathy there was a nominally significant quantitative interaction with a p-value of 0.0013 due to an inconsistent pattern of treatment effect (U-shaped pattern with lowest HRs of 0.45 and 0.42 in patients without MetS and those fulfilling 4–5 criteria compared to HRs of 0.81 and 0.71 in those fulfilling 2 or 3 criteria, respectively).

### Effects on metabolic outcomes

**Fig. 4:**
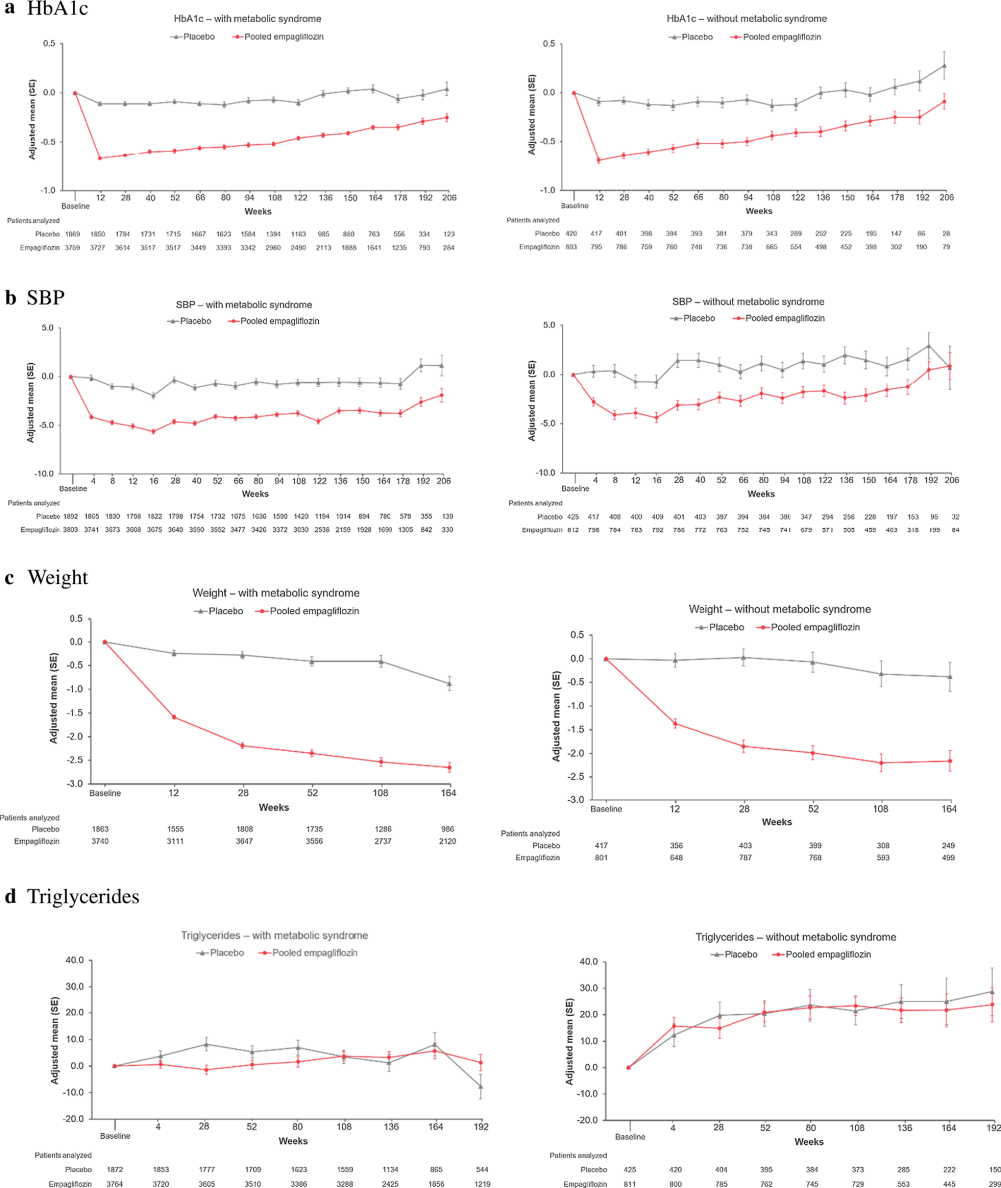

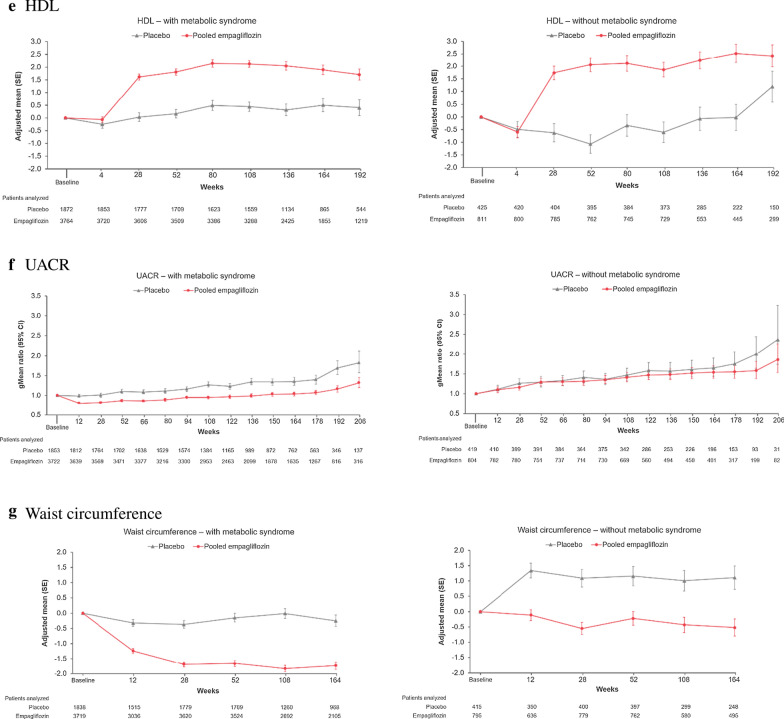
Change from baseline in metabolic outcomes in those with (left panel) vs without (right panel) metabolic syndrome at baseline: **a** HbA1c, **b** SBP, **c** weight, **d** triglycerides, **e** HDL, **f** log(UACR), **g** waist circumference. Results from MMRM models as described in "Materials and methods" section. *HbA1c* glycated hemoglobin, *HDL-C* high-density lipoprotein, *SBP* systolic blood pressure, *UACR* urine albumin creatinine ratio

Empagliflozin reduced HbA1c, SBP, weight, and WC similarly in those with and without metabolic syndrome (Fig. 4). There was a small increase in HDL-C with empagliflozin in both those with or without MetS, whereas triglycerides were not clinically relevantly changed by empagliflozin. UACR was reduced in patients with MetS, whereas in those without MetS, UACR hardly changed over time in any treatment group.

## Discussion

The current analyses show that the majority of the patients included in the EMPA-REG OUTCOME trial fulfilled the WHO criteria for MetS. This is not surprising since MetS is a risk factor for T2D as well as CV disease, both of which constituted inclusion criteria for the trial. Those participants with MetS in fact were at higher risk of adverse CV outcomes. Nonetheless, empagliflozin reduced CV and HF events as well as new or worsening nephropathy irrespective of the MetS status. Importantly, empagliflozin reduced many components of the MetS over time, including WC, SBP, weight, and UACR (only triglycerides were not reduced). Each of these may be viewed as positive cardiometabolic effects of empagliflozin—some of which could conceivably contribute to the SGLT2 inhibitor’s cardiorenal benefits.

Metabolic syndrome is a clustering of interrelated metabolic factors that are associated with increased risk of CV disease and mortality [9]. Insulin resistance/T2D are incorporated in the definition of MetS and, accordingly, patients with T2D have a high prevalence of the MetS. Moreover, patients with T2D have an additive poorer prognosis when multiple components of the MetS are concomitantly present [10–12]. Our study supports that the MetS has prognostic implications in patients with T2D and established ASCVD. In EMPA-REG OUTCOME, patients with both T2D and MetS had a 1.5- to 3-fold higher incidence of cardiorenal outcomes including CV mortality. In particular the relative risk of new or worsening nephropathy was high among those with the MetS. This finding is supported by a previous study showing that the MetS was associated with an increased risk of albuminuria progression and worsening of renal function [13]. Moreover, a report from the Dallas Heart Study demonstrated that both the MetS and T2D were independently associated with higher atherosclerotic burden, evaluated by the content of coronary artery calcium and abdominal aortic atherosclerotic plaques [14]. We have also found that the presence of the MetS was associated with an increased risk of HF hospitalization. The association of the MetS with HF has been reported in other studies and insulin resistance/T2D likely play a major role in HF progression, supporting our findings [15–19].

The consistent treatment effect of empagliflozin on mortality and cardiorenal and HF outcomes regardless of the MetS status expands previous findings demonstrating consistent CV benefit across various subgroups. For example, it has been shown that empagliflozin reduced the risk of cardiorenal and mortality outcomes across subgroups based on the TIMI secondary prevention risk score as well as in those with or without HF and presumed resistant hypertension, and heart failure with reduced ejection fraction irrespective of diabetes and chronic kidney disease [5, 20–23]. Findings, that may be generalized to a large proportion of “real-world” patients [24].

The improvement of the individual components of the MetS over time in patients treated with empagliflozin is clinically relevant, as uncontrolled but potentially modifiable MetS risk factors (e.g., blood pressure, HbA1c, cholesterol, and albuminuria) are incrementally associated with adverse outcomes, and patients with T2D with an optimal risk-factor control might have a similar risk of adverse CV outcomes as people of the same sex and age without T2D [3]. Thus, our findings of an incremental risk in patients with T2D and MetS may increase clinicians´ awareness to identify MetS components in their patients. This is important, as, beyond reducing morbidity and mortality, empagliflozin may also improve most of the MetS components (e.g., glycemic control, blood pressure, weight, and albuminuria) and may also reduce left ventricular mass [25].

### Limitations

Our study has some limitations. These are post-hoc data and thus only hypothesis generating. Furthermore, since the population studied in the EMPA-REG OUTCOME trial had ASCVD we do not know if our results might extend to a general, lower risk T2D population. Finally, the large majority of the trial’s patients had MetS, limiting the statistical power in any comparisons to the non-MetS group.

## Conclusions

A major proportion of the EMPA-REG OUTCOME population fulfills the criteria for MetS. Those with MetS have increased risk of adverse cardio-renal outcomes. Compared with placebo, empagliflozin improved the individual components of the MetS, as well as mortality and cardio-renal outcomes in patients with and without MetS.

## Data Availability

The sponsor of the EMPA-REG OUTCOME Trial (Boehringer Ingelheim) is committed to responsible sharing of clinical study reports, related clinical documents, and patient level clinical study data. Researchers are invited to submit inquiries via the following website: https://trials.boehringer-ingelheim.com.

## Abbreviations

ASCVD: Atherosclerotic cardiovascular disease
BMI: Body mass index
BP: Blood pressure
CV: Cardiovascular
eGFR: Estimated glomerular filtration rate
HF: Heart failure
HR: Hazard ratio
MDRD: Modification of Diet in Renal Disease
MetS: Metabolic syndrome
MI: Myocardial infarction
MMRM: Mixed model repeated measurement
T2D: Type 2 diabetes mellitus
UACR: Urinary albumin-creatinine ratio
WC: Waist circumference
WHO: World Health Organization
3P-MACE: 3-point major adverse cardiovascular events
